# Eating Behaviors of Autistic Women with an Eating Disorder

**DOI:** 10.3390/nu17101622

**Published:** 2025-05-09

**Authors:** Sabrina Schröder, Annemarie van Elburg, Annelies Spek, Unna Danner

**Affiliations:** 1 Altrecht Eating Disorders Rintveld, 3705 Zeist, The Netherlands; s.schroder@altrecht.nl (S.S.); a.a.vanelburg@uu.nl (A.v.E.); 2 Department of Clinical Psychology, Utrecht University, 3584 Utrecht, The Netherlands; 3 Autism Expertise Centrum, 3755 Eemnes, The Netherlands; a.spek@autismeexpertise.nl

**Keywords:** autism spectrum disorder, eating disorders, disordered eating, sensory processing, ARFID

## Abstract

**Background**: Autistic women with eating disorders (EDs) often present with more complex EDs and may not fully benefit from current treatments, yet the reasons for this remain unclear. This study aims to examine the eating behaviors of autistic women with EDs and how these differ from those of (1) non-autistic women with EDs, (2) autistic women without EDs, and (3) non-autistic female controls. It investigates autism-related eating behaviors, traditionally disordered eating behaviors, and avoidant–restrictive food intake disorder (ARFID)-related behaviors to better understand their complex ED presentations. **Methods**: A cross-sectional study was conducted with 30 autistic women with EDs, 30 non-autistic women with EDs, 29 autistic women without EDs, and 60 non-autistic female controls. Participants completed questionnaires assessing eating behaviors, quality of life, and comorbid psychological symptoms. **Results**: Autistic women with EDs exhibited higher levels of both autism-related and disordered eating behaviors than all other groups, including food selectivity, mealtime rigidity, and sensory-related eating difficulties. They also reported notable weight and shape concerns. Additionally, they showed higher levels of comorbidity and reported lower mental health-related quality of life compared to all other groups. **Conclusions**: These findings suggest that the overlap of autism-related and disordered eating behaviors contributes to the complexity and severity of EDs in autistic women, potentially limiting the effectiveness of current treatment approaches. Developing autism-informed interventions that address sensory sensitivities, rigidity, and cognitive differences may improve treatment outcomes. Future research should explore how these factors interact in maintaining ED pathology and identify strategies to distinguish adaptive from maladaptive eating behaviors.

## 1. Introduction

The treatment of eating disorders (EDs) is inherently challenging. Recent studies have suggested that the presence of autism spectrum disorder (ASD) exacerbates treatment complexity, although the underlying reasons remain unclear [1,2]. Problematic eating behaviors are well-documented in autistic* (While we acknowledge that individual preferences vary across autistic communities, this paper adopts “identity-first” language (i.e., “autistic person”) rather than “person-first” language (i.e., “person with autism”) in line with the preferred terminology of the English-speaking autism community [3,4]. Notably, in the Netherlands—the country of origin of the authors and participants—“person-first” language is preferred over “identify-first” language [5]) individuals, but far less is known about eating behaviors in those with a co-occurring ED diagnosis. This knowledge gap is particularly concerning, as this comorbidity appears to contribute to a more complex clinical presentation of EDs, which may, in turn, warrant more tailored treatment approaches to address the specific needs of those with comorbid autism [2,6].

The complexity may, in part, be linked to the high prevalence of distinct feeding and eating behaviors observed in autistic individuals, which themselves are associated with an increased risk of developing feeding and eating disorders [7]. These behaviors can include food selectivity (e.g., eating a restricted range of foods), food neophobia (e.g., avoiding unfamiliar foods), meal-related rigidity (e.g., insistence on using specific utensils), and atypical dietary intake (e.g., excessive fluid consumption) [8,9]. While some of these eating patterns may serve adaptive functions, such as managing sensory sensitivities or alleviating social challenges, they can also increase the risk of nutritional deficiencies and other health complications (e.g., weight problems, type II diabetes, and cardiovascular issues) [10,11,12]. Collectively referred to as autism-related eating behaviors** (While we recognize that eating behaviors such as food neophobia or selectivity are not exclusive to autistic individuals, we use the term ‘autism-related eating behaviors’ to enhance readability and emphasize their markedly higher prevalence in autistic individuals compared to those with other developmental conditions such as ADHD or intellectual disabilities [13,14]), these eating patterns are thought to arise from core autism-related characteristics, including altered sensory processing, differences in social communication, and a heightened need for sameness, structure, and predictability [15,16]. Notably, several of these behaviors overlap with symptoms of avoidant–restrictive food-intake disorder (ARFID), which is increasingly recognized as being particularly common in autistic individuals [8].

Autistic women in particular report high levels of autism-related eating behaviors alongside greater engagement in traditionally disordered eating behaviors [7], and these may be linked to their heightened sensory sensitivity [17,18], greater cognitive and behavioral rigidity [19], increased difficulties with emotion regulation [20], and heightened challenges in social situations involving food [21]. These eating behaviors may include restricting certain food groups (e.g., avoiding high-carbohydrate or high-sugar foods due to fears of weight gain), binge eating, and purging behaviors, placing them at greater risk for developing EDs [18,22]. To help conceptualize the interplay between autism traits and disordered eating, Brede et al. [23] proposed a model in which sensory sensitivities, cognitive rigidity, and social–emotional difficulties are thought to directly and indirectly contribute to the development and maintenance of disordered eating behaviors in autistic women. For example, heightened sensory sensitivity may lead to avoidance of certain textures or temperatures, which over time could contribute to restrictive eating patterns. While our study focuses on these autism-related traits, it is important to note that other contextual factors—such as traumatic experiences or masking autistic characteristics as a way of coping with neurotypical social demands (“camouflaging”)—may also contribute to disordered eating in autistic individuals, as such behaviors may serve as coping strategies, emotional distractions, or arise when cognitive and emotional resources are depleted [24,25].

Once established, EDs in autistic women often present with greater complexity and severity, longer illness duration, poorer daily functioning, and worse long-term psychological outcomes than EDs in non-autistic women [1,2]. Their treatment is suggested to frequently involve intensive interventions, such as prolonged inpatient stays or tube feeding, and autistic women frequently report negative experiences with ED treatments [2,6].

To develop treatments tailored to the unique needs of autistic women with EDs, it is crucial to gain further insight into their specific eating behaviors. The often more complex and severe clinical presentation of EDs in this group may stem from an interplay between autism-related and traditionally disordered eating behaviors, making it difficult to distinguish adaptive from maladaptive patterns [26,27]. Current treatment protocols, such as cognitive behavioral therapy for EDs (CBT-E; [28,29]) do not account for the co-occurrence of autism-related and traditionally disordered eating behaviors and primarily target weight and shape concerns—key drivers of disordered eating behaviors in non-autistic women [30,31]. However, recent research suggests these concerns may play a lesser role in autistic women, who more often attribute their eating difficulties to autism-related challenges, such as sensory sensitivities or cognitive and behavioral rigidity [30,31]. This mismatch may contribute to poorer treatment outcomes in this population [2,6]. Understanding the unique eating behaviors of autistic women is therefore essential to inform the development of more targeted, autism-informed interventions.

In addition to treatment mismatches, further challenges arise from how autism is defined and operationalized in ED research. Many studies rely on samples of women with autistic traits, subthreshold autism, or suspected autism [2]. While research including individuals across the autism spectrum increases inclusivity, it also raises the risk of misattributing common personality traits or psychiatric symptoms to autism, despite their presence in other conditions such as schizophrenia or obsessive–compulsive disorder [32]. Given that an autism diagnosis requires the presence of interrelated core symptoms rather than isolated traits, distinguishing autism from other psychiatric conditions is essential to accurately understand eating behaviors in autistic women. Without studies focusing specifically on formally diagnosed autistic women, it remains unclear whether their eating behaviors align with or differ from those of non-autistic women with an ED. Formally diagnosed samples are therefore essential to ensure that observed eating behaviors are truly autism-specific, rather than reflective of overlapping features of other psychiatric conditions. For example, social withdrawal in individuals with anorexia nervosa may resemble autistic social difficulties but can stem from body-related shame or a desire to avoid eating in front of others, rather than from autism itself [33].

In response to Sharp’s [34] call for high-quality research on eating disorders in autistic individuals, this study aims to investigate the eating behaviors of autistic women with EDs. It compares their behaviors to those of (1) non-autistic women with EDs, (2) autistic women without (lifetime) EDs, and (3) non-autistic women with EDs. Using a set of questionnaires, this study examines a broad range of eating behaviors, including autism-related eating behaviors, traditionally disordered eating behaviors, and eating behaviors often observed in people with ARFID. We hypothesize that autistic women with EDs will report (1) more autism-related eating behaviors, (2) more traditionally disordered eating behaviors, and (3) more ARFID-related eating behaviors (including more picky eating) compared to non-autistic women with EDs, autistic women without EDs, and controls.

## 2. Materials and Methods

### 2.1. Study Design

This study employed a cross-sectional design with a single time-point measurement. Participants completed several questionnaires assessing various eating behaviors using Qualtrics software (Versions 2020–2023; Copyright © 2024 Qualtrics). The study was approved by the Medical Research Ethics Committee in Utrecht, the Netherlands (NL74635.041.20), and by the Institutional Review Board of the Altrecht Mental Health Institute. Additionally, it was pre-registered with the Overview of Medical Research in the Netherlands (OMON; Overzicht van medisch-wetenschappelijk onderzoek in Nederland; NL-OMON20463).

### 2.2. Participants

The sample size was determined based on both statistical considerations and recruitment feasibility. Given the exploratory nature of the study and the limited prior data on comparable populations, a formal power analysis indicated that group sizes of approximately 30 participants would allow detection of medium effect sizes (Cohen’s *d* = 0.36 or larger) with 80% power at an alpha level of 0.05. Feasibility estimates were based on annual patient flows at the participating centers and suggested that the proposed group sizes were realistic within the study’s time frame, including the more difficult-to-recruit ASD + ED group, which required additional diagnostic assessment.

A total of 149 women participated in the study, divided into four groups: autistic women with EDs (ASD + ED group, *n* = 30), non-autistic women with EDs (ED group, *n* = 30), autistic women without EDs (ASD group, *n* = 29), and non-autistic female controls (control group, *n* = 60), matched for age. Participants in the ASD + ED, ED, and ASD groups were recruited from two centers in the Netherlands: Altrecht Eating Disorders Rintveld, a specialized center for the assessment and treatment of EDs (ASD + ED and ED groups), and the Autism Expertise Centrum, a center specialized in the assessment and treatment of autism spectrum disorders (ASD group). Control participants were recruited via Utrecht University. All participants were 18 years of age or older. Before inclusion, they received a letter containing detailed information about the study and provided written informed consent.

### 2.3. Inclusion and Exclusion Criteria

Potential participants were not included if they had a level of education below basic primary education, a mental disability, or insufficient proficiency in Dutch. No participants were excluded based on these criteria.

#### 2.3.1. ASD + ED and ED Groups

To be included in the ASD + ED and ED groups, participants had to meet the DSM-5 diagnostic criteria for an ED [35]. Participants in the ASD + ED group required an additional confirmed autism spectrum disorder diagnosis. Altrecht Eating Disorders Rintveld does not provide treatment for individuals with binge-eating disorder (BED) or pica; therefore, participants with these EDs were not included. Those diagnosed with an unspecified feeding or eating disorder (UFED) were also excluded, as this is a temporary diagnosis at Rintveld. ARFID patients were not included due to the limited number of adults with this diagnosis at the center. Patients with ARFID were not included because the present study focused specifically on autistic women who exhibit both autism-related eating behaviors and traditionally defined ED symptoms such as weight and shape concerns. Including individuals with ARFID, whose restrictive eating is typically not driven by such concerns, would have limited our ability to examine this specific interaction. To maintain a clear distinction between autistic and non-autistic participants, individuals in the ED group who exhibited autistic traits, as measured by the Autism Spectrum Quotient (AQ-50; [36]), were excluded. The ASD + ED group consisted of participants diagnosed with anorexia nervosa (AN) binge–purge type (*n* = 4), AN restrictive type (*n* = 14), bulimia nervosa (BN; *n* = 3), and other specified feeding and eating disorders (OSFED; *n* = 9). The ED group consisted of participants with AN binge–purge type (*n* = 3), AN restrictive type (*n* = 12), BN (*n* = 7), and OSFED (*n* = 8).

#### 2.3.2. ASD Group

Participants in the ASD group were eligible for inclusion if they had an autism spectrum disorder diagnosis according to the DSM-5 criteria [35]. Those with a current or past eating disorder were excluded.

#### 2.3.3. Control Group

Control participants were included if they had no history of psychiatric illness, as determined by the M.I.N.I International Neuropsychiatric Interview [37], and had never been diagnosed with autism spectrum disorder. Those who exhibited autistic traits, as measured by the AQ-50, were excluded.

### 2.4. Procedure

#### 2.4.1. ASD + ED Group and ED Group (Altrecht Eating Disorders Rintveld)

Participants at Rintveld underwent standard diagnostic procedures, including the Eating Disorder Examination interview (EDE; [38]), a psychiatric interview, a medical examination, and a heteroanamnesis with a caregiver or close contact. These assessments were conducted by experienced clinicians (psychiatrists and clinical psychologists), who established ED diagnoses. Autism diagnoses were determined using a screening procedure, regardless of whether the participant had a prior autism diagnosis. The screening included clinician behavioral observations and three diagnostic questions assessing socio-communicative development, social–emotional reciprocity, and cognitive and behavioral flexibility. These questions were answered by both the participant and a caregiver. Screening results were categorized as positive (indicating possible autism), negative (no indication of autism), or unclear. Participants with a positive or unclear screening result underwent a full autism diagnostic assessment. This assessment followed the Dutch Multidisciplinary Guidelines for Diagnostics and Treatment of Autism Spectrum Disorders in Adults [39] and included a semi-structured diagnostic interview, a developmental anamnesis, and a heteroanamnesis, conducted by trained clinicians. Participants who screened negative for autism completed the AQ-50. Those in the ED group who scored above the AQ-50 cut-off of 112 [40] were excluded from the study. This procedure ensured a rigorous and careful diagnostic process for both autism and ED diagnoses, allowing for an accurate classification of participants.

#### 2.4.2. ASD Group (Autism Expertise Centrum)

Participants in the ASD group underwent a similar diagnostic procedure at the Autism Expertise Centrum. To screen for EDs, they completed the SCOFF Questionnaire [41] and additional ARFID-related questions based on the DSM-5 criteria [35]. If necessary, the Structured Clinical Interview for DSM-5 (SCID-5-CV; [42]) was administered. Those with a lifetime ED diagnosis were excluded.

Once diagnoses were confirmed, all participants completed the same online assessment battery. Data collected included demographic information (age and years of education) and clinical characteristics (age of ED onset, illness duration, and age of autism diagnosis), as well as self-reported height and weight measurements, by means of which the body mass index (BMI) was later calculated.

### 2.5. Instruments

Six validated instruments were used to assess eating behaviors, quality of life for people with mental health problems, and clinically relevant psychological symptoms.

The Dutch version of the Eating Disorder Examination Questionnaire (EDEQ; [43,44]) was used to measure eating disorder pathology. This measure consists of 28 items with a variety of different answer possibilities, such as Likert scales and open-ended questions, asking about the frequency with which a patient engages in behaviors indicative of an eating disorder over a 28-day period. There is a global score as well as four subscales: Restraint, Eating Concern, Shape Concern, and Weight Concern. Higher scores indicate greater eating pathology. All Cronbach’s alpha scores were good: *α* > 0.88.

To measure the level of eating and mealtime problems in autistic individuals without an intellectual disability (i.e., autism-related eating behaviors), a Dutch translation of the Swedish Eating Assessment for Autism Spectrum Disorders (SWEAA; [45]) was employed. This measure consists of 60 items comprising 8 subscales, 2 single items, and 5 autism-specific items, as well as demographic and medical background questions, by means of a five-point Likert scale. Scores are added to subscores within their respective subscale: Perception, Motor Control, Purchase of Food, Eating Behavior, Mealtime Surroundings, Social Situation at Mealtime, Other Behavior Associated with Disturbed Eating, and Hunger/Satiety. The questionnaire also contains two single-item subscales, Simultaneous Capacity and Pica. Most Cronbach’s alphas were acceptable or good: *α* > 0.73, except for the Social Situation at Mealtime subscale (*α* = 0.63), which was excluded from further analyses.

A Dutch translation of the Adult Picky Eating Questionnaire (APEQ; [46]) was used to measure picky eating behavior in adults. This multidimensional measure of adult picky eating attitudes and behaviors consists of 16 items and can be answered by means of a five-point Likert scale. There is a global score and four subscales, with higher scores indicating greater adult picky eating: Meal Presentation, Food Variety, Meal Disengagement, and Taste Aversion. Most Cronbach’s alphas were good: *α* > 0.70, except for the Taste Aversion subscale (*α* = 0.50), which was subsequently excluded from further analyses.

To measure the level of ARFID symptomatology, a Dutch translation of the Nine-Item ARFID Screen (NIAS; [47]) was used. This brief multidimensional construct consists of 9 items, which are answered by means of a six-point Likert scale. Items are divided into three subscores, Fear, Lack of Interest, and Picky Eating, from which a global score can be calculated. Higher scores reflect more ARFID symptoms. All Cronbach’s alphas were good: *α* > 0.88.

The Mental Health Quality of Life questionnaire (MHQoL; [48]) is a measure of quality of life specifically developed for use in people with mental health problems. The MHQoL consists of two parts: the MHQoL-7D scale and a corresponding visual analog scale. In this study, only the MHQoL-7D scale was used. It comprises seven questions pertaining to seven dimensions (self-image, independence, mood, relationships, daily activities, physical health, and future), each with four response levels (e.g., ranging from very satisfied to very dissatisfied). Higher scores on the MHQoL-7D indicate better quality of life. The Cronbach’s alpha was good: *α* = 0.86.

The Dutch version of the Brief Symptom Inventory (BSI; [49,50]) was used to assess comorbid psychological symptoms. The BSI consists of 53 items and comprises nine different symptom dimensions: somatic complaints, cognitive problems, interpersonal sensitivity, depressed mood, anxiety, hostility, phobic anxiety, paranoid thoughts, and psychoticism. Items are answered by means of four-point Likert scales (e.g., ranging from not at all to very much), with higher scores indicating more severe symptoms. In the present study, only the BSI total score was used. Additionally, a global severity index (GSI; [49]) was calculated, which is considered to be the most sensitive indicator of the participant’s level of distress as it combines information about the number of symptoms and the intensity of distress. On top of that, the number of present symptoms on the BSI was counted, which is referred to as the number of positive symptoms (PTS). Finally, the severity of the symptoms was determined using the Positive Symptom Distress Index (PSDI). The reliability of the BSI total scale in the present study was excellent: *α* = 0.97.

### 2.6. Data Analysis

All questionnaire data were complete, with no missing responses across participants or measures. Therefore, no imputation procedures were required. Statistical analyses were conducted using IBM SPSS Statistics (Version 29). Univariate ANOVAs and independent-sample *t*-tests were used to compare demographic and clinical characteristics between groups. Univariate ANOVAs were also used to compare outcomes on eating behavior questionnaires, with Tukey’s post hoc pairwise comparisons employed to examine significant group differences. Given the exploratory nature of the study, the modest sample size, and the conceptual overlap across several outcome measures, no correction for multiple comparisons was applied. All results, including effect sizes, are reported to support the interpretation.

## 3. Results

### 3.1. Demographics and Clinical Characteristics

Table 1 presents the demographic and clinical characteristics of each group. Participants in the ASD group were significantly older than those in the ASD + ED group and had completed more years of education. As expected, both the ASD + ED and ED groups had significantly lower BMI values than the ASD and control groups. No significant differences were observed between the ASD + ED and ED groups regarding eating disorder onset or illness duration. Participants in the ASD group received their autism diagnoses significantly later in life than those in the ASD + ED group. Results from the BSI revealed that participants in the ASD + ED group reported higher levels of clinically relevant psychological symptoms (BSI total score), greater psychological distress (BSI global severity index), increased severity of psychological symptoms (BSI PSDI), and a greater number of reported symptoms (BSI PTS) compared to all other groups. The ASD + ED group also reported significantly lower mental health-related quality of life, as assessed by the MHQoL.

### 3.2. Eating Behaviors

Table 2 presents the outcomes on all eating behavior instruments. 

#### 3.2.1. Eating Pathology—EDEQ

Regarding eating disorder pathology, participants in the ASD + ED group exhibited higher overall eating pathology (EDE-Q global score) than all other groups. They also reported significantly greater concerns regarding weight and shape compared to participants in the other groups.

#### 3.2.2. Autism-Related Eating Behaviors—SWEAA

Participants in the ASD + ED group reported higher levels of autism-related eating and mealtime problems (SWEAA total). Specifically, they exhibited greater food selectivity (SWEAA Eating Behavior), more symptoms commonly associated with “traditional” EDs like bingeing and purging (SWEAA Other Behavior Associated with Disturbed Eating) and a heightened need for mealtimes routines (SWEAA Mealtime Surroundings). In terms of sensory-related eating difficulties, participants in the ASD + ED group reported similar levels of difficulties with the sensory input related to food (SWEAA Perception) and the need for control over food-related purchases (SWEAA Purchase of Food) to those in the ASD group, but significantly higher levels than those in the ED group and controls. In terms of being able to do two things simultaneously during a meal (SWEAA Simultaneous Capacity), both the ASD + ED and ASD groups experienced greater difficulties than the ED group and controls. Across all diagnostic groups, similar difficulties were reported in recognizing sensations of hunger and satiety, with higher scores than those observed in controls. No significant group differences were found in the presence of pica-related behaviors.

#### 3.2.3. Adult Picky Eating Behaviors and Attitudes—APEQ

Participants in the ASD + ED group exhibited significantly elevated overall picky eating (APEQ global score) compared to all other groups. They also displayed greater rigidity regarding meal presentation and preparation (APEQ Meal Presentation), increased food neophobia (APEQ Food Variety), and more pronounced issues with mealtime disengagement and avoidance (APEQ Meal Disengagement) than all other participants. Additionally, the ASD + ED group demonstrated a greater tendency to reject bitter and sour foods (APEQ Taste Aversion) compared to the ED group and controls, though their scores did not differ significantly from those of the ASD group.

#### 3.2.4. ARFID-Associated Eating Behaviors—NIAS

Participants in the ASD + ED group showed elevated ARFID symptomatology (NIAS global score), particularly greater fear of choking, vomiting, or gastrointestinal distress (NIAS Fear). All three diagnostic groups (ASD + ED, ED, and ASD) had similar levels of food avoidance due to sensory sensitivities (NIAS Picky Eating), which were significantly higher than those observed in the control group. Finally, participants in both the ASD + ED and ED groups reported comparable patterns of food avoidance or reduced intake due to a lack of appetite or interest (NIAS Lack of Interest), with scores significantly higher than those in the ASD and control groups.

## 4. Discussion

The findings of this study provide important insights into the eating behaviors of autistic women with EDs, highlighting both shared and unique patterns compared to non-autistic women with EDs, autistic women without EDs, and non-autistic controls. In line with our hypotheses, autistic women with EDs reported (1) significantly higher levels of autism-related eating behaviors, (2) more traditionally disordered eating behaviors, and (3) elevated ARFID-related symptoms and picky eating compared to all other groups. Specifically, they experienced more food-related sensory difficulties, food selectivity, meal-related rigidity, food neophobia, and disengagement from and avoidance of mealtimes. While their interoceptive challenges (e.g., recognizing hunger/satiety cues) were similar to those of non-autistic women with EDs, they were more impaired than autistic women without EDs. Notably, autism-related eating behaviors were significantly more prevalent in autistic women with EDs than in autistic women without EDs. These findings align with those of Brede et al. [51], who found that autistic women with restrictive EDs also displayed higher levels of autism-related eating behaviors than both non-autistic women with restrictive EDs and autistic women without EDs. According to the authors, this suggests that such autism-related eating behaviors are not merely general features of autism but are, in fact, characteristic of the subgroup of autistic women who develop EDs.

Contrary to prior suggestions, this study found that autistic women with EDs exhibited not only elevated disordered eating behaviors, but also greater weight and shape concerns than all other groups [30,31]. These findings challenge the assumption that weight and shape concerns play a lesser role in the development and maintenance of EDs in autistic women. They also contrast with the findings of Brede et al. [51], who reported lower levels of disordered eating and fewer weight and shape concerns in autistic women with restrictive EDs compared to their non-autistic counterparts. A potential explanation is that Brede et al. [51] grouped autistic women with ARFID and (atypical) AN, meaning some exhibited restrictive eating not driven by weight and shape concerns. One possible explanation for the heightened weight and shape concerns in our study is that autistic women may view their weight and shape as a means of social integration. Previous qualitative research suggests that some autistic women focus on their weight and shape as a way to conform to neurotypical social norms [23,52]. Additionally, cognitive styles common in autism, such as literal thinking and black-and-white reasoning, may exacerbate these concerns, reinforcing rigid perceptions of what to weigh and how to look (e.g., “If I’m not thin, then I’m fat and horrible”) [23,53]. However, it is important to note that the Dutch validation study of the EDE-Q [43] did not support the instrument’s theorized four-factor structure, including the subscales for weight and shape concern. This limitation warrants caution in interpreting these subscales. Nevertheless, given the assumption that autistic women with EDs experience weight and shape concerns differently, our findings underline the need for further research to critically assess these beliefs and clarify their role in this population.

Our results confirm clinical and empirical observations that ED presentations in autistic women tend to be particularly severe and complex [2,6]. This complexity appears to stem from the interplay of high levels of autism-related and disordered eating behaviors, where both adaptive (e.g., food selectivity to manage sensory issues) and maladaptive behaviors (e.g., food restriction due to weight and shape concerns) are simultaneously present. The co-occurrence of ARFID symptoms, such as a fear of choking or gastrointestinal distress, further complicates this clinical picture. This overlap poses significant clinical challenges. On the one hand, some autism-related eating behaviors may be mistakenly pathologized as disordered eating behaviors. On the other, ED pathology may be misattributed to autistic characteristics, leading to potential misdirection in treatment [27]. Moreover, findings on the BSI and MHQoL suggest that autistic women with EDs experience particularly severe psychological distress, with higher levels of comorbid symptoms and a significantly lower mental health-related quality of life than all other groups.

The findings highlight the pressing need for tailored treatment approaches that consider the unique eating behaviors observed in autistic women with EDs. Standard ED treatments, such as CBT-E, may require modifications to accommodate altered sensory processing, differences in information processing, rigid thinking styles, and differences in social communication. A critical component of autism-informed ED care is distinguishing adaptive eating behaviors (e.g., food selectivity to manage sensory sensitivities or difficulties with novelty) from maladaptive behaviors that contribute to ED pathology (e.g., restriction due to weight concerns). Developing additional treatment modules focused on this distinction could help clinicians validate and support beneficial eating strategies while effectively addressing harmful behaviors such as bingeing, purging, and extreme dietary restriction. In this context, clinicians should remain cautious not to over-attribute eating behaviors to autism alone, as weight and shape concerns might remain a significant factor in this group despite previous assumptions. While some autistic women with EDs may benefit from adapted interventions—such as sensory-informed strategies or modifications to cognitive–behavioral approaches—clinical experience also shows that others respond well to standard treatments without the need for adjustments. This subgroup may have lower levels of sensory sensitivity or greater cognitive flexibility, which could reduce the need for modifications. Additionally, some may prioritize weight and shape concerns over autism-related challenges, making traditional ED treatment targets possibly more relevant and effective. Recognizing this variability is essential in tailoring care without assuming that all autistic individuals require fundamentally different approaches.

Beyond identifying adaptive and maladaptive behaviors, practical modifications to ED treatment may help autistic women engage more effectively in therapy. These could include providing a structured and predictable treatment environment, adapting therapy pace to accommodate information processing differences, or offering sensory-informed nutritional rehabilitation, taking into account individual sensory profiles [54]. By implementing these modifications and creating an autism-friendly treatment environment, clinicians may improve treatment accessibility and outcomes for autistic women with EDs. Future research should explore how such autism-adapted interventions impact treatment efficacy and long-term recovery trajectories. Additionally, ensuring participatory approaches in treatment development is crucial [21]. Autistic individuals need to be actively involved in discussions on how to incorporate autism-specific factors into therapy, particularly sensory sensitivities and cognitive rigidity. Moreover, their perspectives could provide valuable insights into how weight and shape concerns should be interpreted and addressed in treatment, ensuring that interventions align with their lived experiences and clinical needs.

## 5. Strengths, Limitations, and Future Directions

This study has several methodological strengths, including thorough diagnostic assessments to confirm autism diagnoses and the inclusion of both autistic and non-autistic control groups. However, several limitations should be acknowledged. First, height and weight data were self-reported rather than clinically measured. While this approach ensured consistency across all groups, it may have introduced bias. The use of self-report measures may have also influenced how participants interpreted questionnaire items, making it difficult to determine whether certain behaviors reflect autism-related behaviors or disordered eating behaviors. Second, while our sample size was adequate for the planned group comparisons, it was not sufficient to conduct covariate-adjusted or mediation analyses, nor to explore differences between ED subtypes within groups. Although our inclusion of various ED subtypes reflects the clinical reality that comorbid autism occurs across ED diagnoses, it is likely that additional factors—such as ED subtype and symptom severity—may further shape the eating behaviors of autistic women with EDs. The current study was not designed to examine such differences, and this represents an important avenue for future research. Relatedly, we did not control for potentially confounding variables such as age and education, which may have influenced the findings. Future research should investigate whether age-related factors—such as increased life experience and improved coping mechanisms—help buffer against the development of disordered eating in autistic individuals without EDs. Third, although we observed similar picky eating patterns across the three diagnostic groups, this study was not designed to disentangle whether these behaviors are autism- or ED-driven, nor to identify the underlying mechanisms linking sensory sensitivities, ARFID symptoms, and traditional ED pathology. We also did not include individuals with ARFID to focus on autistic women with traditional ED profiles. While this was a deliberate decision, it limits the conclusions about the overlap between ARFID symptoms and autism-related eating behaviors. Additionally, given that cultural context may shape eating behaviors and weight and shape concerns, the use of a Dutch-only sample may limit generalizability and partially may explain differences from studies conducted in other countries, such as Brede et al.’s [51]. Finally, as multiple statistical comparisons were conducted without formal correction, there is a risk of type I error. However, this decision was based on the exploratory aim of the study and the interdependence of the measures, which made conservative corrections, such as Bonferroni correction, inappropriate due to the risk of overlooking meaningful effects.

Future research may want to investigate eating behaviors and potential mechanisms underlying weight and shape concerns in autistic women with various subtypes of EDs and consider how autistic characteristics (such as altered sensory processing or differences in thinking styles) and comorbid symptoms interact in the development and maintenance of ED pathology [16,51]. Additionally, future work should explore how overlapping or co-occurring symptoms (e.g., ARFID symptoms and autism-related eating behaviors) interact with traditionally disordered ED behaviors and influence treatment response. Investigating whether sensory accommodations in treatment may inadvertently reinforce restrictive eating would also be valuable. Finally, research should evaluate how specific treatment adaptations—such as sensory-informed interventions or modifications to CBT-E—can be matched to individual profiles, supporting a flexible, personalized approach to care.

## 6. Conclusions

This study highlights the complex and multifaceted nature of ED presentations in autistic women, emphasizing both shared and distinct features, compared to non-autistic women with EDs and autistic women without EDs. The findings challenge previous assumptions about the limited role of weight and shape concerns and underscore the importance of tailored interventions that address both autism-related and traditionally disordered eating behaviors. By developing strategies to distinguish between adaptive and maladaptive behaviors and fostering a more autism-friendly treatment environment, clinicians and researchers can improve treatment effectiveness and better meet the needs of this population.

## Figures and Tables

**Table 1:** Means (and SDs) of descriptive variables: demographic and clinical characteristics per group (ED group, ED + ASD group, ASD group, and control women), as well as group differences.

	ED + ASD (*n* = 30)		ED (*n* = 30)		ASD (*n* = 29)		Controls (*n =* 60)					
	Mean	*SD*	Mean	*SD*	Mean	*SD*	Mean	*SD*	*F*_3,145_	*p*	η_p_^2^	*t*
Age	24.23 ^c^	7.36	31.13	13.29	34.72 ^a^	12.11	29.40	9.71	5.01 *	0.002	0.09	
Years of education	13.63 ^c^	3.30	15.92	2.77	17.00 ^a,d^	3.85	16.10 ^c^	3.76	5.08 *	0.002	0.10	
BMI	18.82 ^c,d^	3.92	18.78 ^c,d^	2.93	24.36 ^a,b^	4.24	23.20 ^a,b^	3.36	21.88 **	<0.001	0.31	
Age of onset of ED	16.60	4.45	18.63	6.54	-	-	-	-	-	0.164	-	1.41
Illness duration of ED (years)	7.63	6.21	12.50	13.05	-	-	-	-	-	0.007	-	1.84
Age of autism diagnosis	20.97 ^c^	8.12	-	-	33.10 ^a^	11.62	-	-	-	0.037	-	4.64 **
BSI Total	55.17 ^b,c,d^	31.33	30.03 ^a,d^	19.71	19.72 ^a,d^	20.97	5.20 ^a,b,c^	8.45	44.54 **	<0.001	0.48	
BSI GSI	1.04 ^b,c,d^	0.59	0.57 ^a,d^	0.37	0.37 ^a,d^	0.40	0.10 ^a,b,c^	0.16	44.54 **	<0.001	0.48	
BSI PST	28.10 ^b,c,d^	11.90	18.07 ^a,c,d^	9.11	11.86 ^a,b,d^	9.75	3.57 ^a,b,c^	5.25	58.10 **	<0.001	0.55	
BSI PSDI	1.83 ^b,c,d^	0.43	1.54 ^a^	0.32	1.46 ^a^	0.39	1.36 ^a^	0.43	8.34 **	<0.001	0.17	
MHQoL	8.30 ^b,c,d^	3.10	10.43 ^a,d^	2.97	12.41 ^a,d^	3.31	15.92 ^a,b,c^	2.79	50.46 **	<0.001	0.51	

* p < 0.05, ** *p* < 0.001. BMI, body mass index. BSI Total, Brief Symptom Inventory Total Score. BSI GSI, Brief Symptom Inventory Global Severity Index. BSI PST, Brief Symptom Inventory Positive Symptom Total. BSI PSDI, Brief Symptom Inventory Positive Symptom Distress Index. ED, eating disorder. MHQoL, Mental Health Quality of Life Index. ^a^ Different from ED + ASD group. ^b^ Different from ED group. ^c^ Different from ASD group. ^d^ Different from control group.

**Table 2:** Means (and SDs) of eating behavior variables per group (ED + ASD group, ED group, ASD group, and control women), as well as group differences.

		ED + ASD (*n* = 30)		ED (*n* = 30)		ASD (*n* = 29)		Controls (*n =* 60)				
		Mean	*SD*	Mean	*SD*	Mean	*SD*	Mean	*SD*	*F*_3,145_	*p*	η_p_^2^
EDEQ	Global	4.22 ^b,c,d^	1.12	3.39 ^a,c,d^	1.36	1.06 ^a,b^	0.86	0.74 ^a,b^	0.72	111.63 **	<0.001	0.70
	Restraint	3.83 ^c,d^	1.46	3.22 ^c,d^	1.58	0.72 ^a,b^	0.90	0.62 ^a,b^	0.92	70.65 **	<0.001	0.59
	Eating concern	3.43 ^c,d^	1.26	2.81 ^c,d^	1.33	0.57 ^a,b^	0.72	0.25 ^a,b^	0.46	109.64 **	<0.001	0.69
	Shape concern	4.99 ^b,c,d^	1.06	4.04 ^a,c,d^	1.58	1.69 ^a,b^	1.40	1.16 ^a,b^	1.14	77.90 **	<0.001	0.62
	Weight concern	4.62 ^b,c,d^	1.33	3.5 ^a,c,d^	1.56	1.25 ^a,b^	1.11	0.94 ^a,b^	1.11	73.19 **	<0.001	0.60
SWEAA	Global	163.93 ^b,c,d^	34.11	124.50 ^a,d^	26.90	136.38 ^a,d^	31.36	91.67 ^a,b,c^	15.05	57.68 **	<0.001	0.54
	Perception	32.50 ^b,d^	9.98	20.83 ^a,c^	6.53	29.86 ^b,d^	9.60	17.90 ^a,c^	5.84	31.51 **	<0.001	0.40
	Motor control	12.03	3.98	10.23 ^c^	3.55	13.34 ^b,d^	4.06	10.42 ^c^	2.75	6.10 **	<0.001	0.11
	Purchase of food	11.43 ^b,d^	2.60	8.60 ^a,d^	2.88	9.69 ^d^	3.31	6.58 ^a,b,c^	2.52	22.46 **	<0.001	0.32
	Eating behavior	17.87 ^b,c,d^	5.71	14.43 ^a,d^	5.07	13.90 ^a,d^	5.12	8.82 ^a,b,c^	2.73	31.01 **	<0.001	0.39
	Mealtime surroundings	35.80 ^b,c,d^	9.20	24.40 ^a,d^	9.07	25.21 ^a,d^	10.14	13.42 ^a,b,c^	3.07	61.79 **	<0.001	0.56
	Social situations at mealtime	25.43 ^d^	4.76	22.50 ^d^	4.76	24.83 ^d^	4.33	19.85 ^a,b,c^	4.43	13.49 **	<0.001	0.22
	Other behaviors associated with disturbed eating	18.67 ^b,c,d^	5.41	15.30 ^a,c,d^	4.22	10.45 ^a,b^	2.57	9.07 ^a,b^	1.58	62.78 **	<0.001	0.57
	Hunger/satiety	6.57 ^d^	1.50	5.60 ^d^	1.98	5.69 ^d^	1.75	3.40 ^a,b,c^	2.04	33.22 **	<0.001	0.41
	Simultaneous capacity	2.63 ^b,d^	1.33	1.50 ^a,c^	0.90	2.38 ^b,d^	1.24	1.22 ^a,c^	0.49	19.72 **	<0.001	0.29
	Pica	1.00	0.00	1.10	0.55	1.03	0.19	1.00	0.00	1.14	0.337	0.02
APEQ	Global	2.95 ^b,c,d^	0.78	2.18 ^a,d^	0.50	2.24 ^a,d^	0.62	1.67 ^a,b,c^	0.46	35.24 **	<0.001	0.42
	Meal presentation	3.02 ^b,c,d^	0.85	2.32 ^a,d^	0.47	2.44 ^a,d^	0.67	1.78 ^a,b,c^	0.54	27.09 **	<0.001	0.36
	Food variety	2.83 ^b,c,d^	0.65	1.97 ^a,d^	0.74	2.38 ^a,d^	0.67	1.55 ^a,b,c^	0.53	31.37 **	<0.001	0.39
	Meal disengagement	2.78 ^b,c,d^	1.08	2.06 ^a,d^	0.80	2.18 ^a,d^	0.78	1.58 ^a,b,c^	0.66	15.02 **	<0.001	0.24
	Taste aversion	3.20 ^b,d^	1.03	2.27 ^a,d^	0.83	2.79 ^d^	0.93	1.70 ^a,b,c^	0.67	25.17 **	<0.001	0.34
NIAS	Global	21.43 ^b,c,d^	9.58	15.4 ^a,d^	9.45	13.79 ^a,d^	8.89	4.58 ^a,b,c^	5.36	33.83 **	<0.001	0.41
	Picky eating	6.43 ^d^	3.78	4.77 ^d^	3.27	6.69 ^d^	4.50	2.40 ^a,b,c^	2.83	14.21 **	<0.001	0.23
	Lack of interest	8.37 ^c,d^	3.66	6.63 ^c,d^	3.61	4.34 ^a,b,d^	3.98	1.42 ^a,b,c^	2.37	36.20 **	<0.001	0.43
	Fear	6.63 ^b,c,d^	4.64	4.00 ^a,d^	4.16	2.78 ^a,d^	3.27	0.77 ^a,b,c^	1.70	21.95 **	<0.001	0.31

** *p* < 0.001. ^a^ Different from ED + ASD group. ^b^ Different from ED group. ^c^ Different from ASD group. ^d^ Different from control group.

## Data Availability

The raw data supporting the conclusions of this article will be made available by the authors on reasonable request.

## Abbreviations

The following abbreviations are used in this manuscript:

APEQ	Adult Picky Eating Questionnaire
ARFID	Avoidant–restrictive food intake disorder
BMI	Body mass index
BSI GSI	Brief Symptom Inventory Global Severity Index
BSI PSDI	Brief Symptom Inventory Positive Symptom Distress Index
BSI PST	Brief Symptom Inventory Positive Symptom Total
BSI TOT	Brief Symptom Inventory Total Score
ED	Eating disorder
EDEQ	Eating Disorder Examination Questionnaire
MHQoL	Mental Health Quality of Life
SWEAA	Swedish Eating Assessment for Autism Spectrum Disorders
